# Flush Flow Behaviour Affected by the Morphology of Intravascular Endoscope: A Numerical Simulation and Experimental Study

**DOI:** 10.3389/fphys.2021.733767

**Published:** 2021-11-19

**Authors:** Yujie Li, Mingzi Zhang, Simon Tupin, Kohei Mitsuzuka, Toshio Nakayama, Hitomi Anzai, Makoto Ohta

**Affiliations:** ^1^Institute of Fluid Science, Tohoku University, Sendai, Japan; ^2^Centre of Health Research, Torrens University Australia, Pyrmont, NSW, Australia; ^3^Faculty of Medicine, Health, and Human Sciences, Macquarie Medical School, Macquarie University, Sydney, NSW, Australia; ^4^Graduate School of Biomedical Engineering, Tohoku University, Sendai, Japan; ^5^Nara College, National Institute of Technology, Yamatokoriyama, Japan

**Keywords:** intravascular endoscope, haemodynamics, computational fluid dynamics, volume fraction, multiphase flow, *in vitro* flow experiment

## Abstract

**Background:** Whilst intravascular endoscopy can be used to identify lesions and assess the deployment of endovascular devices, it requires temporary blockage of the local blood flow during observation, posing a serious risk of ischaemia.

**Objective:** To aid the design of a novel flow-blockage-free intravascular endoscope, we explored changes in the haemodynamic behaviour of the flush flow with respect to the flow injection speed and the system design.

**Methods:** We first constructed the computational models for three candidate endoscope designs (*i.e.*, Model A, B, and C). Using each of the three endoscopes, flow patterns in the target vessels (straight, bent, and twisted) under three different sets of boundary conditions (*i.e.*, injection speed of the flush flow and the background blood flowrate) were then resolved through use of computational fluid dynamics and *in vitro* flow experiments. The design of endoscope and its optimal operating condition were evaluated in terms of the volume fraction within the vascular segment of interest, as well as the percentage of high-volume-fraction area (PHVFA) corresponding to three cross-sectional planes distal to the microcatheter tip.

**Results:** With a mild narrowing at the endoscope neck, Model B exhibited the highest PHVFA, irrespective of location of the cross-sectional plane, compared with Models A and C which, respectively, had no narrowing and a moderate narrowing. The greatest difference in the PHVFA between the three models was observed on the cross-sectional plane 2 mm distal to the tip of the microcatheter (Model B: 33% vs. Model A: 18%). The background blood flowrate was found to have a strong impact on the resulting volume fraction of the flush flow close to the vascular wall, with the greatest difference being 44% (Model A).

**Conclusion:** We found that the haemodynamic performance of endoscope Model B outperformed that of Models A and C, as it generated a flush flow that occupied the largest volume within the vascular segment of interest, suggesting that the endoscope design with a diameter narrowing of 30% at the endoscope neck might yield images of a better quality.

## Introduction

With the clinical need for more accurate diagnosis of intravascular lesions, intravascular endoscopy can be used to instantaneously visualise the lesions *in vivo* and thus assess the severity of disease (Ueda et al., 2004; Sun, 2013; Horie, 2021). Furthermore, intravascular endoscopy can also be used to examine the structure of endovascular devices after deployment, thereby evaluating the effectiveness of treatment (Jang et al., 2002; Vardar et al., 2009; Xu and Sun, 2015).

Although recent developments in optical imaging have markedly improved the performance of endoscope system (Fujimoto et al., 2000; Boese et al., 2017), the presence of the surrounding blood flow remains a major hurdle for intravascular endoscopes to produce a clear and undistorted view of the target lesion. Current intravascular endoscopy available on the market still requires temporary blockage of the background blood flow with a balloon during observation or treatment, which can pose a serious risk of ischaemia especially when used in the coronary arteries (Li Q. et al., 2018).

Recently, a novel design of intravascular endoscope has been proposed to avoid the potential risk of ischaemia while obtaining clear images of the target lesion. The mechanism is to generate a transparent volume by high-speed injection of a limpid fluid (e.g., destran or saline) through the microcatheter, to allow the endoscope camera to capture clear images of the target lesion (Nerandzic et al., 2021). Simple theories of fluid mechanics suggest that the flush flow behaviours would be affected by a variety of factors, including the flow injection speed, the shape of the flush flow channel, and the condition of the background blood flow.

To aid in the optimal design of such a flow-blockage-free intravascular endoscope, we aimed to examine the flush flow behaviours corresponding to three types of endoscope prototypes under a variety of operating conditions. We first constructed the computational models of the candidate endoscope designs, and then resolved the flow patterns when the flush was injected into the target vessel through use of computational fluid dynamics (CFD) and *in vitro* flow experiments. We hypothesised that a higher volume fraction of the flush flow would contribute to generation of clearer images of the target lesion.

## Materials and Methods

### Computational Fluid Dynamics Simulation of Flush Flow Behaviour

#### Endoscope System and Flow Channel in the Blood Vessel

**FIGURE 1:**
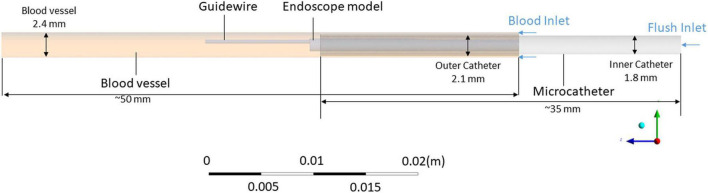
3D model of the flow channel for simulation of the blood and flush flow behaviour.

Since the left anterior descending artery was the coronary artery where stenosis most commonly occurred, this simulation aimed to mimic the endoscope examination in this vessel. As shown in Figure 1, the simulation model for the endoscope system consists of an endoscope, a microcatheter, a guidewire, and a segment of blood vessel. Two inlets were defined, respectively, for the background blood flow (the primary phase) and the flush flow (the secondary phase), and an outlet was defined for the mixture of the two phases. The diameter of the blood vessel was set as 2.5 mm, which is within the range of the common size of the target artery (Zafar et al., 2014).

#### Intravascular Endoscope Models

**FIGURE 2:**
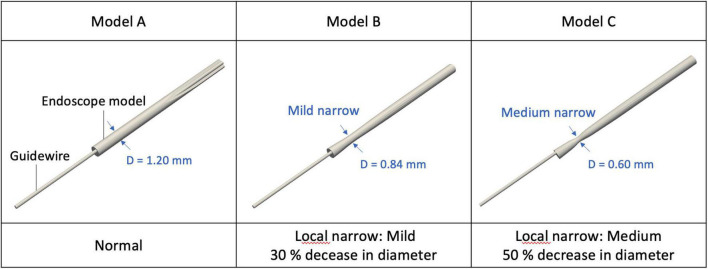
3D simulation model of endoscope with different morphological characteristics.

The shape of the flush flow channel located between the endoscope and the microcatheter varies among different designs of the endoscope. Any morphological alteration of the endoscope will affect the flow pattern of the flush flow. In the present study, three endoscope models (Model A, B, and C) with different morphological characteristics were created for simulations, as shown in Figure 2. Per the structure of the endoscope prototype, a narrowing was created for Models B and C at the endoscope neck, respectively, with a 30% and 50% reduction in diameter. Compared with Model A, such a morphological variation created an expansion in the flow channel for flush flow.

#### Computational Fluid Dynamics Simulation

For each endoscope system, a predominantly tetrahedral computational grid was created for the fluid zone using a commercial software tool, ICEM-CFD (Ansys, United States), with three prismatic layers adhering to the vascular wall. To ensure simulation accuracy, the quality and robustness of the computational grid was checked by a mesh dependency test, which revealed that the mesh with a total of 7.2 million elements would be adequate for a reliable simulation.

Multiphase flow simulations were performed for all cases. The blood flow was defined as the primary phase and the flush flow as the secondary phase. The density and viscosity of blood were, respectively, specified as 1050 kg/m^3^ and 0.0035 Pa^⋅^s. To mimic the properties of dextran, the density and viscosity of flush were set as 1080 kg/m^3^ and 0.0043 Pa^⋅^s. The blood and flush were assumed to be an incompressible Newtonian fluid (Li Y. et al., 2018). A constant boundary condition was set for the flush flow at 180 ml/min. Three typical boundary conditions were assumed for background blood flow, respectively, at 25, 50, and 100 ml/min (hereinafter referred to as BC I, II, and III), that cover the typical range of blood flowrate in human coronary artery (Spiller et al., 1983; Anderson et al., 2000; Aarnoudse et al., 2007; Zafar et al., 2014). The surfaces of the blood vessel, the endoscope, and the catheter were assumed to be rigid. The flow simulation was performed using Fluent (Ansys, United States) based on a finite-volume method.

#### Haemodynamic Characteristics

Two-dimensional velocity vectors and contours, together with 3D velocity iso-surfaces were generated to visualise the flush flow behaviour, in terms of the velocity, flow direction, recirculation, *etc*.

Volume fraction contours of the flush flow were generated on three cross-sectional observation planes, respectively, located 2, 5, and 10 mm distal to the microcatheter tip. Associations of the volume fraction with the background blood flow and the morphology of endoscope were also quantified.

Since a greater volume fraction close to the vascular walls would be critical for the camera to take a clear picture of the arterial lesions, volume fraction of the flush flow at the 30% and 50% outer torus areas on each cross-sectional plane were, respectively, analysed in this study.

The percentage of high-volume-fraction area (PHVFA) was defined as ratio of the area on a cross-sectional plane with a volume fraction >80% to the area of the entire cross-sectional plane. We calculated the PHVFA corresponding to the three observation planes and their respective 30% and 50% outer torus.

### Physical Flow Experiment to Observe the Delivery Ratio of the Flush Fluid

#### Experimental Flow System

**FIGURE 3:**
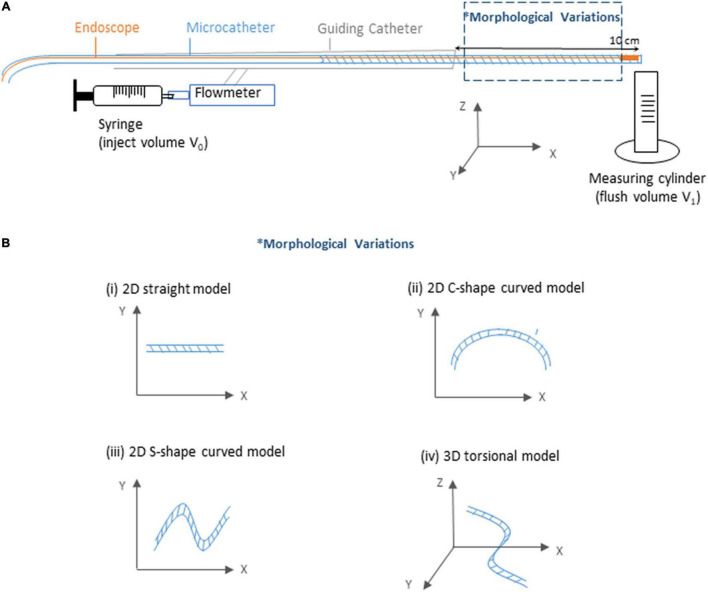
Sketch of the experimental flow and measurement system. **(A)** Sketch of the experiment platform. **(B)** Sketch of the morphological variations of the microcatheter.

As show in Figure 3A, an experiment platform was established to measure the flush transportation within the endoscope system, which consisted of three parts: the endoscope, the microcatheter to create a flow channel for the flush flow, and the guiding catheter.

For brevity, the flush fluid was prepared using distilled water as a substitute of dextran, as this does not affect exploration of the volume fraction of the flush flow. To inject the flush fluid, a syringe was connected to the guiding catheter via a Y-connector, with a flowmeter deployed to measure the injection speed. In the distal end of the endoscope system, a measuring tube was prepared to measure the volume of the outflow (V_1_). With a certain amount of flush fluid (V_0_, 15 ml) injected into the endoscope system every time across all experiment cases, the ratio of flush fluid delivery was then calculated by the ratio between V_1_ and V_0_.

#### Experiment Model

Since coronary lesions may occur at any location of the coronary artery (Xu and Sun, 2015), it is important that the endoscope system is flexible enough to travel through bent and twisted pathways. To investigate the potential impacts of complicated vascular morphologies on the flush flow delivery efficacy, we tested four types of microcatheter morphology — straight tube, 2D curved tubes, respectively, with a “C” shape (2D-C) and a “S” shape (2D-S), and 3D torsional tube, as shown in Figure 3B.

#### Flow Characteristics

At a variety of injection speeds, the delivery ratios of flush flow (V_1_/V_0_) from injection to the outlet of the fluid channel within the microcatheter were calculated, for various shapes of the microcatheter.

## Results

### 2D Velocity Vector Field Affected by the Morphology of Endoscope

**FIGURE 4:**
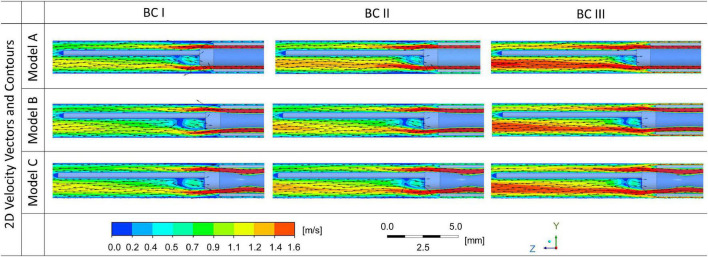
2D velocity vectors and contours of flush flow affected by the morphologies of endoscope models and flowrates of the background blood flow.

2D velocity vectors and contours of flush flow in the middle plane along the axial direction of the blood vessel were generated, for cases with endoscope systems in three different morphologies, at three different blood flow conditions, as shown in Figure 4.

While the velocity magnitude and distribution remained similar between cases with endoscope Model A, B, and C, the background blood flowrate was found to have a stronger impact on the velocity magnitude and distribution of flush flow. Endoscope operating condition at BC III – the highest background blood flowrate - created the highest velocity for the flush flow.

According to the 2D velocity vectors, the flow pattern of flush flow in the blood vessel showed tiny differences between Model A, B, and C. The recirculation flow, which occurred around the guidewire just after the tip of the microcatheter, showed slight difference – the size of the recirculation decreased as the flowrate of blood flow increased.

### Velocity of Flush Flow Affected by the Morphology of Endoscope

**FIGURE 5:**
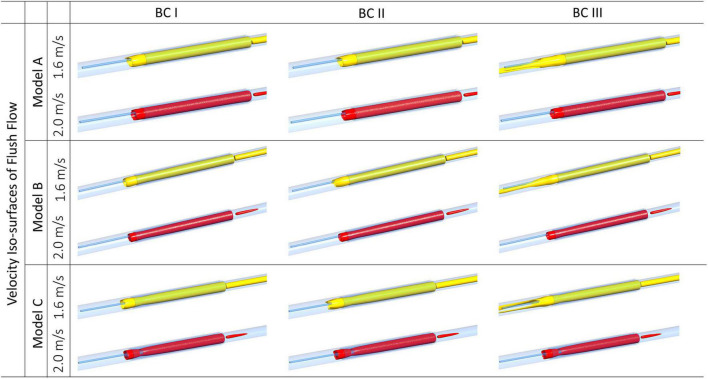
Velocity iso-surfaces of flush flow affected by endoscope morphologies and flowrates of the background blood flow.

As shown in Figure 5, iso-surfaces of velocities at 1.6 m/s and 2.0 m/s were, respectively, generated for Model A, B, and C, under different flowrates of background blood flow. Iso-surfaces at velocity 2.0 m/s remained similar across different cases, whereas iso-surfaces at velocity 1.6 m/s showed apparent difference between BC III and others. Compared to BC I and II, endoscope operating condition at BC III created extended high-velocity streams at both sides of the guidewire along the axial direction.

### Volume Fraction of Flush Flow at Different Cross-Sectional Planes

**FIGURE 6:**
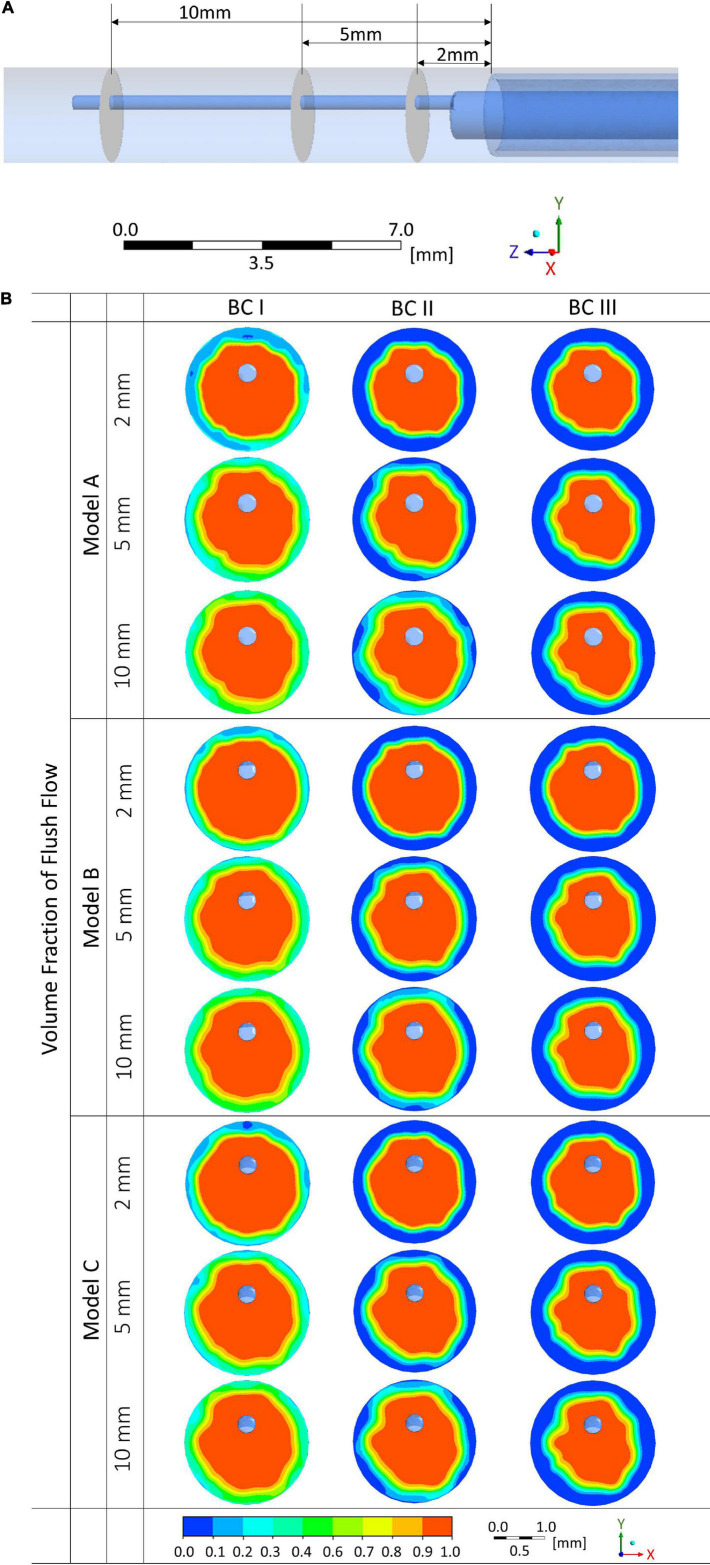
Volume fractions of flush flow affected by endoscope morphologies and flowrates of the background blood flow at different cross-sectional planes. **(A)** Locations of the cross-sectional planes. **(B)** Volume fraction contours of the flush flow.

Volume fraction of flush flow were examined at three cross-sectional planes in the blood vessel at the downstream of the endoscope system, locations of the planes are displayed in Figure 6A, which are, respectively, at 2, 5, and 10 mm distal to the microcatheter tip.

Comparing between results from Models A, B, and C, differences in the distribution of volume fraction were not significant. However, the flowrate of background blood flow created a marked difference. As shown in Figure 6B, at simulation scenario of BC I, volume fraction of flush higher than 50% occupies most of the space on the cross-sectional planes, while at simulation conditions of BC II and III, low volume fraction of flush can be observed around the vascular wall. Moreover, differences in volume fraction can also be noted between the three cross-sectional planes. Plane at 10 mm distal to the microcatheter tip revealed the highest volume fractions of flush, followed by planes at 5 mm and 2 mm downstream.

### Quantitative Analysis of the Volume Fraction

**FIGURE 7:**
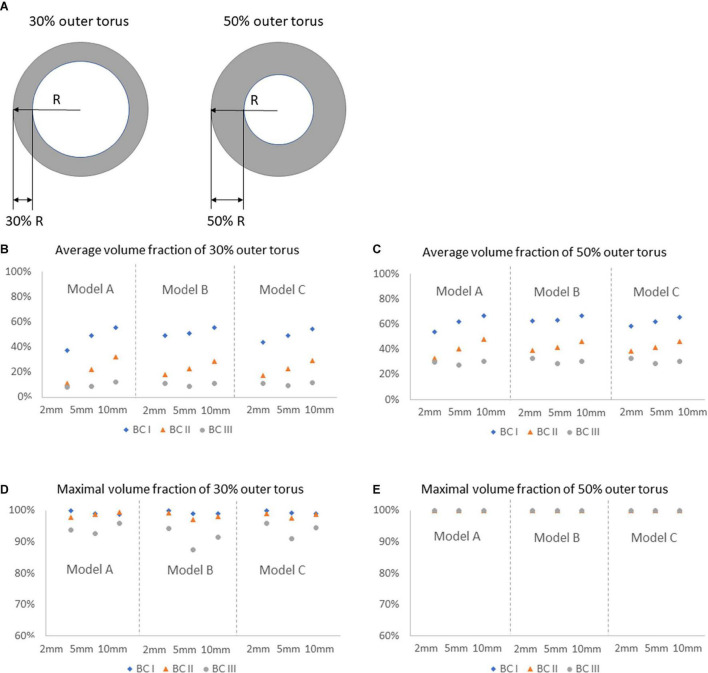
Comparison of average and maximal volume fractions of flush flow on different cross-sectional planes, in 30 and 50% outer torus, under three flowrates of the background blood flow, for Models A, B, and C. **(A)** Sketch of the 30 and 50% outer torus. **(B)** Average volume fraction of 30% outer torus. **(C)** Average volume fraction of 50% outer torus. **(D)** Maximal volume fraction of 30% outer torus. **(E)** Maximal volume fraction of 50% outer torus.

Comparisons of average and maximal volume fractions of flush flow between Model A, B, and C were carried out, on different cross-sectional planes and the corresponding 30% and 50% outer torus areas, under three blood flow environments, as reported in Figure 7. The sketches of 30% and 50% outer torus area are shown in Figure 7A. Comparing between Figures 7B,C, the average volume fractions were greater in 50% outer torus than 30% outer torus across all simulation scenarios. This increase was more substantial when the simulation was performed under BC II and III, of approximately 20 percentage points, whereas increase of about 10 percentage points was discovered for simulations under BC I. From Figures 7D,E, it reveals that maximal volume fractions of flush flow in the 30% outer torus are above 85% in all cases, mainly distributed between 90 and 100%, while that in the 50% outer torus reach up to 100%.

**FIGURE 8:**
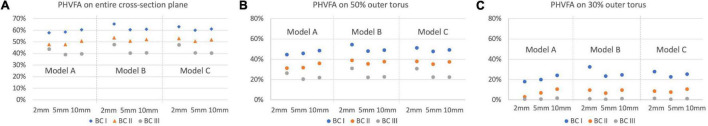
Comparison of the PHVFA of flush flow on different cross-sectional planes and the corresponding 30 and 50% outer torus, under three flowrates of the background blood flow, for Models A, B, and C. **(A)** PHVFA on the entire cross-sectional plane. **(B)** PHVFA on 50% outer torus. **(C)** PHVFA on 30% outer torus.

Percentage of high-volume-fraction area (PHVFA) of flush flow were further calculated, as shown in Figure 8. On the entire plane, value of PHVFA ranged from 40 to 65% for all conditions (see Figure 8A), while on 50% outer torus the values decreased to a range of 21 to 54% (see Figure 8B) and further decreased to less than 33% on the 30% outer torus (see Figure 8C). Importantly, a difference in PHVFA caused by the morphological characteristics between Model A, B and C was observed. Across all comparisons between the corresponding simulation cases, Model B had a relatively greater PHVFA, closely followed by Model C, while Model A has the smallest values. The greatest difference in the PHVFA between the three models was observed on the cross-sectional plane 2 mm distal to the tip of the microcatheter (Model B: 33% vs. Model A: 18%), while the average difference between them was about 7%.

### Influence of Catheter Morphology on the Delivery of Flush Flow

According to the *in vitro* flow experiment, the successful flush delivery ratios from injection to the outlet of the endoscope system were calculated, for microcatheter in different morphologies (see Figure 3B), at a variety of injecting speeds, as shown in Table 1.

**TABLE 1:** Flush delivery ratios for experiment cases with various microcatheter morphologies at different injecting speeds.

Experiment No.	Injecting volume (V_0_, ml) ^1^	Injecting time (s) ^2^	Injecting speed (ml/s) ^3^	Flush volume from catheter tip (V_1_, ml) ^4^	Ratio of delivery ^5^
*Catheter morphology: Straight*					
1	15.0	39.0	0.38	14.5	97%
2	15.0	35.0	0.43	14.5	97%
3	15.0	15.0	1.00	14.0	93%
4	15.0	12.0	1.25	14.0	93%
5	15.0	10.0	1.50	14.0	93%
6	15.0	8.5	1.76	14.0	93%

*Catheter morphology: 2D C-curve*					
7	15.0	30.0	0.50	14.0	93%
8	15.0	25.0	0.60	14.0	93%
9	15.0	15.0	1.00	14.0	93%
10	15.0	12.0	1.25	14.0	93%

*Catheter morphology: 2D S-curve*					
11	15.0	30.0	0.50	14.0	93%
12	15.0	20.0	0.75	14.0	93%
13	15.0	10.0	1.50	14.0	93%

*Catheter morphology: 3D torsion*					
14	15.0	27.0	0.56	14.0	93%
15	15.0	20.0	0.75	14.0	93%
16	15.0	10.0	1.50	13.5	90%

*^1^Injecting volume (V_0_) is the total volume of flush solution prepared in the syringe to be injected into the endoscope system.*

*^2^Injecting time is measured using a stopwatch, from the beginning to the end of a single time injection with constant speed.*

*^3^Injecting speed is calculated with V_0_ and the injecting time, which is the overall velocity of the injection.*

*^4^Flush volume (V_1_) is the total volume of flush flow coming out of the catheter tip, measured by a measuring tube.*

*^5^Ratio of delivery is the ratio between V_1_ and V_0_, which means the successful delivery ratio of flush solution at the treating area.*

When the microcatheter was placed in the straight shape, a series experiments were performed with flush injecting speed increasing from 0.38 to 1.76 ml/s. The corresponding successful delivery ratios were varying from 97 to 93% of the total injected volume. When the microcatheter was placed as 2D C-shape or S-shape curved model, a series experiments were performed with injecting speed increasing from 0.50 to 1.50 ml/s. The successful delivery ratio was found to be consistent at 93%. When the microcatheter was placed in a 3D torsional model, results from experiments with injecting speed increase from 0.56 to 1.50 ml/s indicated a successful delivery ratio ranging from 93 to 90%.

## Discussion

In this study, we performed multiphase computational fluid dynamic simulations to observe the flow pattern and volume fraction of flush flow in the blood vessel, with three various endoscope models, at three flowrates to mimic the background blood environment. *In vitro* flow experiments were also implemented to examine the successful delivery ratio of flush fluid when the endoscope system travelled through some bent and twisted pathways before reaching the diseased area. Results of this study demonstrates the potential of this type of endoscope system to realise the visualisation of luminal blood vessel, and the influence on the volume fraction of flush caused by different factors.

### Influence on Flush Flow Behaviour by Morphological Characteristics of Endoscope

Three endoscope models with morphological variations were considered in this study. Model A had a uniform diameter along the axial direction of the endoscope, while Model B and C had thinner necks with, respectively, 30% and 50% less diameter, which changed the flow channel of flush flow in the microcatheter with an expansion. Such expansion usually creates alterations in pressure at the expanded section, resulting in variations to the velocity and divergence of the flow streams at the distal end of the expansion, similar as the typical flow pattern in the blood vessel with a fusiform aneurysm.

From the comparison of 2D velocity vectors and contours (Figure 4), flush flow velocities (Figure 5), and volume fractions (Figures 6, 7), there were rarely substantial differences observed between Model A, B, and C. However, differences in PHVFA demonstrated the influence of endoscope morphology on volume fractions (Figure 8). With a mild narrowing at the endoscope neck, Model B exhibited the highest PHVFA, irrespective of location of the cross-sectional plane, compared with Models A and C which, respectively, had no narrowing and a moderate narrowing. It indicates that the endoscope design with a mild narrowing at the endoscope neck might yield images of a better quality. Although such kind of mild morphological variations did not bring substantial haemodynamic changes, more significant morphological variations may yield different disturbing effects in the flush flow patterns.

### Influence on Flush Flow Behaviour by Flowrate of Blood

Influence on flush flow behaviour were compared by velocity vectors and contours, and volume fractions of flush flows, between three blood flow conditions – blood flowrate at 25 (BC I), 50 (BC II), and 100 (BC III) ml/min.

According to Figure 3, when exiting from the micro-catheter and entering the blood vessel, flush flow has the highest velocity at BC III, indicating the velocity of flush flow increases as the flowrate of blood increases.

Moreover, the comparison of volume fractions in Figure 6B shows, when the flowrate of background blood increases from BC I to III, the volume fraction distribution of flush remains almost consistent at the centre of the blood vessel, while decreasing significantly around the blood vessel wall. According to the quantitative analysis, volume fractions at BC III and I revealed an average drop of 39% and 32%, respectively, in the 30% and 50% torus area. This results also indicated that the variation of blood flow condition had a significant effect on the volume fraction of flush flow close to the blood vessel wall.

It should also be noted from Figures 7D,E that the maximal volume fractions of flush flow in the 30% outer torus were above 85% in all cases, mainly distributed between 95 and 100% in cases under BC I and II. This finding indicates a high possibility of using the endoscope system to clearly view the inner wall of the blood vessel.

### Influence on Volume Fraction of Flush Flow by Location

Volume fractions of flush flow at three cross-sectional planes, respectively, located at 2, 5, and 10 mm at the downstream of the microcatheter tip were examined.

As can be seen from Figure 6, volume fractions of flush remain high in the centre area of the blood vessel, at over 90% in all scenarios. From 2 mm to 10 mm cross-sectional planes, the volume fraction of flush increased on 30% and 50% outer torus. This tendency was more notable in cases under BC I and II; for cases under BC III, the volume fractions of flush maintained at low level on the outer torus regardless of the cross-sectional plane location. Cross-sectional plane at the farthest location (examined by this study) showed a relatively well-developed volume fraction of flush. However, it should be considered and further examined that whether the flush flow with this level of volume fraction can be adequate to create a clear view in front of the endoscope camera, to support the assessment of the plaque status.

### Blood Vessel Shape Cause Little Influence on the Delivery of the Flush Flow

To mimic the bending and twisting effects of patient-specific arteries on the shape of the guiding catheter, the guiding catheter were adjusted into four scenarios, straight, 2D-C, 2D-S, and 3D torsional models. The consumption of flush (*i.e.*, the fluid stays in the guiding catheter) during its transportation from the injecting point to the end of the guiding catheter before entering the blood vessel were measured via experiments. The delivery ratio reported in Table 1 shows that, instead of entering the blood vessel, less than 10% of injected fluid may stay in the endoscope system, regardless of the shape of the blood vessel. On the other hand, injecting speed played a more important role in the successful delivery ratio of the flush fluid.

### Influence on the Flush Flow Behaviour by the Unsuccessful Delivery of Flush

**FIGURE 9:**
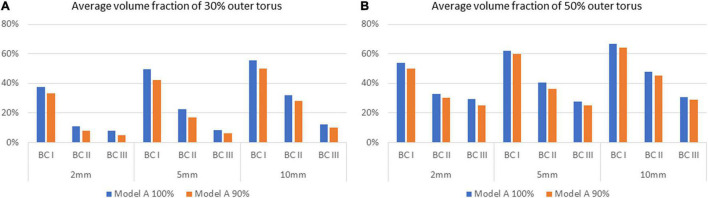
Comparison of average volume fractions of flush flow for Model A between 100% and 90% delivery of the flush fluid, on the 30% and 50% outer torus on different cross-sectional planes, under three flowrates of the background blood flow. **(A)** Average volume fraction of 30% outer torus. **(B)** Average volume fraction of 50% outer torus.

To investigate the effect of unsuccessful delivery of flush obtained from the *in vitro* flow experiments, a separate series of simulation was performed with Model A, with 10% lower inflow rate of flush set as the simulation boundary condition, to compensate the unsuccessful delivery of flush. Comparison of flush flow behaviours was then carried out between the original boundary conditions and the compensated one (respectively, denoted by Model A 100% and 90% in Figure 9), revealing a small drop in volume fraction of flush flow at around 4%.

### Limitations

Some assumptions were adopted in this study. First of all, CFD was implemented using a steady-state assumption, however, it can be reliable as other studies have demonstrated that steady-state models provide reasonable estimates for the time-averaged haemodynamics of true pulsatile flow (Geers et al., 2014). Therefore, an indication of the effect of varying blood flowrate can also be gained by examining a variety of flowrates. Meanwhile, the flowrate of flush flow was set at a constant value of 180 ml/min across all simulation cases, however, it should also be noted that other values may be adopted depending on individual cases. We adopted 180 ml/min in this study as it is one of the common choices in the real operation with endoscope.

Second, in the *in vitro* flow experiment, distilled water was used as a substitute of dextran for the conveniency of operation. However, the use of water was kept through the entire experiment for different scenarios, which neither brought in additional variables nor uncertainties for the evaluation of the flush delivery ratio in guiding catheters.

Moreover, due to the interaction between the geometry of the patient-specific blood vessel and the endoscope system, the treatment scenario varies individually. Besides that, clinicians with different training and experience may have their own habits and decisions in real operations. However, these variations cannot be fully covered by this study. This study aims to provide the preliminary evidence for clinicians as well as researchers to estimate the potential behaviour of the endoscope system.

As a preliminary study, this study was not designed to thoroughly consider a wide range of parameters, including a complete series of model with systematically designed morphological characteristics. It is our future work to establish and study more sophisticated models.

## Conclusion

In the present study, computational models for three prototypes of flow-blockage-free intravascular endoscope were constructed. Corresponding to each model, the volume fraction and PHVFA of the flush flow under three sets of boundary conditions were quantified through use of multiphase computational fluid dynamics simulation.

We found that the haemodynamic performance of endoscope Model B outperformed that of Models A and C, as it generated a flush flow that occupied the largest volume within the vascular segment of interest, suggesting that the endoscope design with a 30% diameter narrowing at the endoscope neck might yield images of a better quality.

## Data Availability Statement

The original contributions presented in the study are included in the article/supplementary material, further inquiries can be directed to the corresponding author.

## Author Contributions

MO, TN, HA, and YL analysed the original concept. MO, YL, and TN directed the research. TN and YL designed CFD experiments. YL, TN, MZ, and KM performed CFD experiments. YL and MZ analysed CFD data and wrote the manuscript. ST, YL, and KM designed flow experiment. YL, ST, and KM performed flow experiments. YL and MO analysed flow experiment data. YL, MZ, HA, and MO revised the manuscript. All authors contributed to the article and approved the submitted version.

## Conflict of Interest

The authors declare that the research was conducted in the absence of any commercial or financial relationships that could be construed as a potential conflict of interest. The handling editor declared a past co-authorship with several of the authors MZ, ST, HA, and MO.

## Publisher’s Note

All claims expressed in this article are solely those of the authors and do not necessarily represent those of their affiliated organizations, or those of the publisher, the editors and the reviewers. Any product that may be evaluated in this article, or claim that may be made by its manufacturer, is not guaranteed or endorsed by the publisher.
